# Involvement of presenilin holoprotein upregulation in calcium dyshomeostasis of Alzheimer's disease

**DOI:** 10.1111/jcmm.12008

**Published:** 2013-02-05

**Authors:** Kamran Honarnejad, Christian KE Jung, Sven Lammich, Thomas Arzberger, Hans Kretzschmar, Jochen Herms

**Affiliations:** a Department of Translational Brain Research, DZNE – German Center for Neurodegenerative DiseasesMunich, Germany; b Center for Neuropathology and Prion Research, Ludwig Maximilian UniversityMunich, Germany; c Graduate School of Systemic Neurosciences, Ludwig Maximilian UniversityMunich, Germany; d Adolf Butenandt Institute – Biochemistry, Ludwig Maximilian UniversityMunich, Germany; e Munich Cluster for Systems Neurology (SyNergy)Munich, Germany

**Keywords:** presenilin, holoprotein, calcium, Alzheimer's disease, endoplasmic reticulum

## Abstract

Mutations in presenilins (PS1 and PS2) account for the vast majority of early onset familial Alzheimer's disease cases. Beside the well investigated role of presenilins as the catalytic unit in γ-secretase complex, their involvement in regulation of intracellular calcium homeostasis has recently come into more focus of Alzheimer's disease research. Here we report that the overexpression of PS1 full-length holoprotein forms, in particular familial Alzheimer's disease-causing forms of PS1, result in significantly attenuated calcium release from thapsigargin- and bradykinin-sensitive stores. Interestingly, treatment of HEK293 cells with γ-secretase inhibitors also leads to decreased amount of calcium release from endoplasmic reticulum (ER) accompanying elevated PS1 holoprotein levels. Similarly, the knockdown of PEN-2 which is associated with deficient PS1 endoproteolysis and accumulation of its holoprotein form also leads to decreased ER calcium release. Notably, we detected enhanced PS1 holoprotein levels also in postmortem brains of patients carrying familial Alzheimer's disease PS1 mutations. Taken together, the conditions in which the amount of full length PS1 holoprotein is increased result in reduction of calcium release from ER. Based on these results, we propose that the disturbed ER calcium homeostasis mediated by the elevation of PS1 holoprotein levels may be a contributing factor to the pathogenesis of Alzheimer's disease.

## Introduction

Alzheimer's disease (AD) is the most common form of adult dementia characterized by the extracellular accumulation of amyloid beta (Aβ) protein and formation of intracellular neurofibrillary tangles leading to neuronal dystrophy and loss [1, 2]. Mutations in presenilins (PS) account for the majority of early onset familial Alzheimer's disease (FAD) cases [3]. Upon undergoing endoproteolytic processing and forming N- and C-terminal fragments (NTF and CTF), PS functions as the catalytic subunit of γ-secretase multiprotein complex on the cell surface, comprising PS, Nicastrin, Aph-1, and PEN-2 [4]. Among over 60 different type I transmembrane protein substrates, γ-secretase sequentially cleaves amyloid precursor protein (APP) after β-secretase cleavage to generate Aβ [5]. FAD mutations in PS have been shown to alter the cleavage of APP in favour of neurotoxic Aβ42 generation [6]. However growing body of evidence also indicates that FAD-PS mutations impair several intracellular calcium signalling mechanisms, particularly endoplasmic reticulum (ER) calcium homeostasis [7, 8]. Uncleaved full-length (FL) PS holoprotein is approximately 50 kD in size and primarily located on the ER membrane [9]. ER calcium store has approximately 1000-fold higher calcium concentration than cytosol [10]. The exact molecular mechanism as to how FAD-PS mutations cause ER calcium dyshomeostasis is not fully resolved. Yet PS have been shown to affect multiple components of ER calcium handling [11]. PS holoprotein has been proposed to form passive calcium leak channel on the ER membrane [12, 13] through its hydrophilic catalytic cavity [14], regulate Inositol 1,4,5-triphosphate (InsP_3_) receptor gating [15, 16], Ryanodine receptor (RyR) channel activity [17, 18] and abundance [19]. PS have also been shown to interact with Sarco/endoplasmic reticulum calcium-ATPase (SERCA) pump which actively transfers calcium from cytosol into the ER [20], and to modulate this function [21, 22]. Capacitative calcium entry (CCE) – the process of refilling intracellular calcium stores through plasma membrane channels – has been shown to be attenuated in cells expressing FAD PS mutants [23, 24]. Moreover, PS2 modulates calcium shuttling between ER and mitochondria [25]. Here we aimed to examine the potential role of impaired PS endoproteolysis leading to accumulation of PS holoprotein on the ER membrane [26, 27] in the context of disrupted ER calcium homeostasis in AD.

## Materials and Methods

### Cell culture and cell lines

Human embryonic kidney 293 (HEK293) cells were cultured in Dulbecco's modified eagle medium (DMEM) supplemented with 10% fetal bovine serum and 1% penicillin/streptomycin while being incubated at 37°C, 5% CO_2_ and 90% humidity. PS1-overexpressing and PEN-2 knockdown HEK293 lines were generously provided by Dr. H. Steiner. HEK293 cells stably expressing either PS1 wild type or mutant variants, or AChRa1 were generated by transfection of HEK293 cells with the respective cDNA cloned into pcDNA3.1/Zeo(+) (Invitrogen, Carlsbad, CA, USA) and subsequent selection for zeocin (100 μg/ml) resistance. Likewise, RNA interference-mediated PEN-2 stable knockdown clone was generated by stable transfection of pSUPER/PEN-2-163 and pcDNA3.1/Hygro(−) (Invitrogen) into HEK293 cells and subsequent selection for hygromycin (100 μg/ml) resistance [28].

### Calcium imaging

One day prior to the experiment, HEK293 cells were plated in a 96-well collagen coated microplate (Greiner BioOne GmbH, Frickenhausen, Germany) at 40,000 cells/well. Cytosolic calcium concentration was measured using the Fluo-4 NW kit (Invitrogen Corporation, Madison, WI, USA) according to manufacture's instructions. Briefly, the growth medium was exchanged with a freshly mixed calcium-free assay buffer. The cells were incubated at 37°C for 30 min., then for an additional 30 min. at room temperature. Fluorimetric calcium measurements were performed utilizing a confocal laser-scanning system (Carl Zeiss AG, Jena, Germany) equipped with a climate control chamber (EMBL, Heidelberg, Germany). Cells were then imaged using a 40× oil immersion objective (Zeiss Plan-Apochromat, Carl Zeiss; 40× NA 1.3). Excitation of the cells was performed at 488 nm with an Argon Laser (Zeiss) and the emission was collected using band pass filter (500–550 nm). Time-lapse fluorescence images were acquired at 5 sec. interval for Thapsigargin (TP; 1 μM) and 1 sec. interval for Bradykinin (BK; 300 nM) and Carbachol (CCh; 10 μM). Subsequently images were analysed by defining typically 20–30 regions of interest (ROI) for individual cells in each well using the Zeiss LSM 510 Meta Software. Data analysis was performed using Microsoft EXCEL (Microsoft, Seattle, WA, USA), Sigma Plot (SPSS, Chicago, IL, USA) and GraphPad Prism 5.0b (GraphPad Software, San Diego, CA, USA). All fluorescence data are expressed as ΔF/F_0_ = (F − F_0_)/F_0_, where F is the measured fluorescence signal at any given time and F_0_ is the average fluorescence from the scans preceding stimulation.

If not otherwise stated, values represent mean ± SEM. To test significance, student's *t*-test (two tailed) was performed and differences were considered statistically significant if *P* < 0.05.

### Treatment with γ-secretase inhibitors

Human embryonic kidney 293 cells were grown to 60–70% confluency inside of 10 cm petri dishes. γ-secretase inhibitors (all from Calbiochem, Darmstadt, Germany) were added to the growth medium and incubated for 24 hrs at concentrations which were reported to inhibit the γ-secretase activity. DAPT, Gamma IV and Gamma XXI were used respectively at 10 μM, 2.7 μM and 300 nM concentration. Controls were treated in parallel with DMSO vehicle instead of inhibitors.

### Western blot

Human embryonic kidney 293 cells were lysed in ‘complete lysis-M buffer’ with protease inhibitor mix (Roche Molecular Biochemicals, Indianapolis, IN, USA) according to the manufacturer's instructions. Similarly for human brain material, a small piece from frozen postmortem frontal cortex of FAD as well as control cases were cut and homogenized in sucrose/hepes buffer with PMSF. Protein concentrations were measured using BCA assay. Equal amounts of protein samples were separated in a 10% tris-glycine SDS-PAGE and transferred to PVDF-membrane (Millipore Corporation, Bedford, MA, USA). For detection of presenilin holoprotein, a rabbit polyclonal antibody against a MBP/PS1-loop (aa 263–407) fusion protein was used at 1:500 dilution (antibody 5023; a kind gift from Dr. H. Steiner [29]). Mouse monoclonal anti-Tubulin (Santa Cruz Biotechnology, Santa Cruz, CA, USA) was used at 1:1000 dilution for loading control and corresponding AP-coupled secondary antibodies (Thermo Scientific, Waltham, MA, USA) at 1:5000 dilution. Chemiluminescent reaction was performed with CDP-Star (Roche Molecular Biochemicals) and detected with a Chemocam Imager (INTAS Science Imaging Instruments GmbH, Göttingen, Germany). Western blot bands were quantified using Advanced Image Data Analyzer/2D Densiotometry 3.52 (Raytest GmbH, Straubenhardt, Germany).

### Human subjects

In total seven frontal cortex samples comprising three FAD-PS1 and one FAD-APP mutation carrying patients as well as three control individuals were collected from BrainNet Europe. The staging of samples was determined according to Braak & Braak during routine postmortem tissue diagnostics by skilled neuropathologists [30]. The use of human tissue samples was approved by the institutional review board of the University of Munich (BrainNet: Brain Banking Center Munich).

## Results

### Effect of PS1 holoprotein overexpression on calcium release from ER

**Fig. 1:**
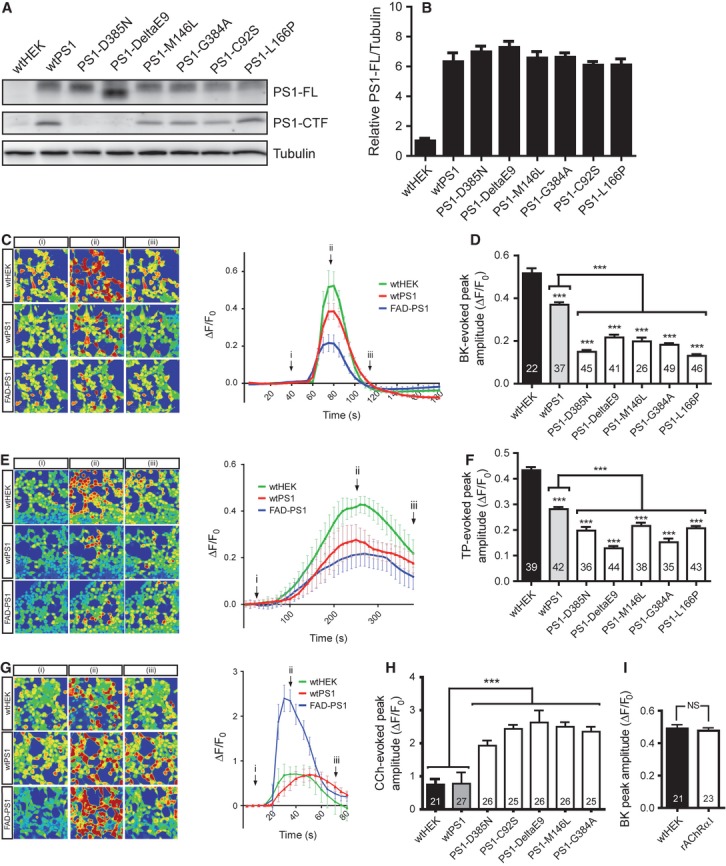
Altered calcium release from ER in PS1-overexpressing HEK293 cells (**A**) Representative immunoblot indicating the expression of PS1 in HEK293 cells. The expression of PS1-FL holoprotein and PS1-CTF protein were analysed using MBP/PS1-loop (aa 263–407) antibody on western blot using the protein lystes from wild type cells, cells stably overexpressing wild type PS1, several FAD-PS1 mutants and a γ-secretase inactive mutant (PS1-D385N). 5 μg of protein lysate was loaded into each lane on the gel. (**B**) Densitometric quantification of PS1-FL band intensity normalized to loading control Tubulin (*n* = 3). (**C**) Representative traces of BK-evoked, (**E**) TP-evoked and (**G**) CCh-evoked calcium releases for wild type HEK293 cells, HEK293 cells stably overexpressing wild type or mutant PS1 and the corresponding calcium images for the time points indicated with arrows displayed in pseudocolors. (**D**) Average peak amplitude of BK-evoked and (**F**) TP-evoked calcium release from ER are significantly reduced in cells stably expressing wild type PS1 *versus* wild type HEK293 cells. Furthermore BK- and TP-evoked calcium responses are significantly attenuated in cells overexpressing mutants form of PS1 in comparison to both wild type PS1-overexpressing and plain wild type HEK293 cells. (**H**) Average peak amplitude of Carbachol (CCh)-evoked calcium release from ER is significantly amplified in HEK293 cells stably expressing mutant forms of PS1 relative to cell stably expressing wild type PS1 and plain wild type HEK293 cells (***P < 0.001). (**I**) Average peak amplitude of BK-evoked calcium release from ER is unchanged in HEK293 cells stably overexpressing rAChRα1 relative to wild type HEK293 cells (NS: Non-significant).

To assess the role of increased PS1 holoprotein levels in the ER calcium homeostasis, we used HEK293 cells stably expressing either wild type or several different mutant forms of PS1. Typically the endogenous PS1 holoprotein level is relatively low, being on the border line of detection [31]. We confirmed remarkable increase in PS1 holoprotein expression level by western blotting protein lystes from PS1 stable lines (Fig. 1A). Densitometric analysis indicate six- to sevenfold increase in the PS1 full length holoprotein levels in all stable clones compared to the wild type HEK293 cells (Fig. 1B). Likewise the PS1-CTF levels were increased in all the clones, except for PS1-DeltaE9 and PS1-D385N which both lack the endoproteolytic cleavage site (Fig. 1A). Overexpression of wild type PS1 and to a higher degree various FAD-PS1 mutants led to significantly lowered calcium release from ER in comparison to the untransfected controls. The ER calcium responses were generated by applying Bradykinin (BK). Application of BK leads to liberation of calcium from InsP_3_-sensitive ER stores. The peak amplitude of the BK-evoked calcium release alone in wild type PS1 (wtPS1) overexpressing cells decreased to 71 ± 2.2% of control wild type HEK293 cells. All FAD-PS1 mutants further lowered the amplitude of BK-evoked calcium release peak size as follows: PS1-DeltaE9 to 41 ± 2.5%, PS1-M146L to 38 ± 3.4%, PS1-G384 to 35 ± 1.2% and PS1-L166P to 25 ± 1.4% of the wild type HEK293 controls (Fig. 1D). These results were confirmed using Thapsigargin (TP) as well. TP is an inhibitor of SERCA pump that blocks calcium uptake into ER, causing the diffusion of calcium from ER into the cytosol due to a very strong calcium gradient. Following a similar trend, the peak amplitude of TP-evoked calcium release in wtPS1 overexpressing cells was reduced to 65 ± 1.9%, in PS1-DeltaE9 to 29 ± 2.1%, in PS1-M146L to 49 ± 2.9%, in PS1-G384 to 35 ± 3.2% and in PS1-L166P to 47 ± 2.1% of the wild type HEK293 controls (Fig. 1F). Importantly, overexpression of a mutant form of PS1 being functionally inactive for γ-secretase substrate cleavage (PS1-D385N) [32], also resulted in lowered BK-evoked (to 28 ± 1.6% of wild type) and TP-evoked (to 45 ± 3.3% of wild type) calcium liberation from ER, indicating that the observed effects are independent of γ-secretase substrate cleavage activity (Fig. 1D and F). Moreover, there was no correlation observed between the PS1-CTF levels and the attenuated calcium response from ER. PS1-DeltaE9 and PS1-D385N mutants, which do not undergo endoproteolysis and thus do not generate PS1-CTF, showed comparable attenuation of ER calcium release as to the rest of FAD-PS1 mutants which generate approximately proportionate levels of PS1-CTF. Conversely, the amplitude of calcium release upon activation of muscarinic receptors with carbachol (CCh) was enhanced in FAD-PS1 mutant and PS1-D385N expressing cells (Fig. 1H). On a note, the overexpression of rat nicotinic acetylcholine receptor subunit alpha 1 (rAChRα1), an unrelated protein which also localizes to the ER did not alter the amplitude of BK-evoked calcium release in HEK293 cells (Fig. 1I).

### Effect of γ-secretase inhibitors on calcium release from ER

**Fig. 2:**
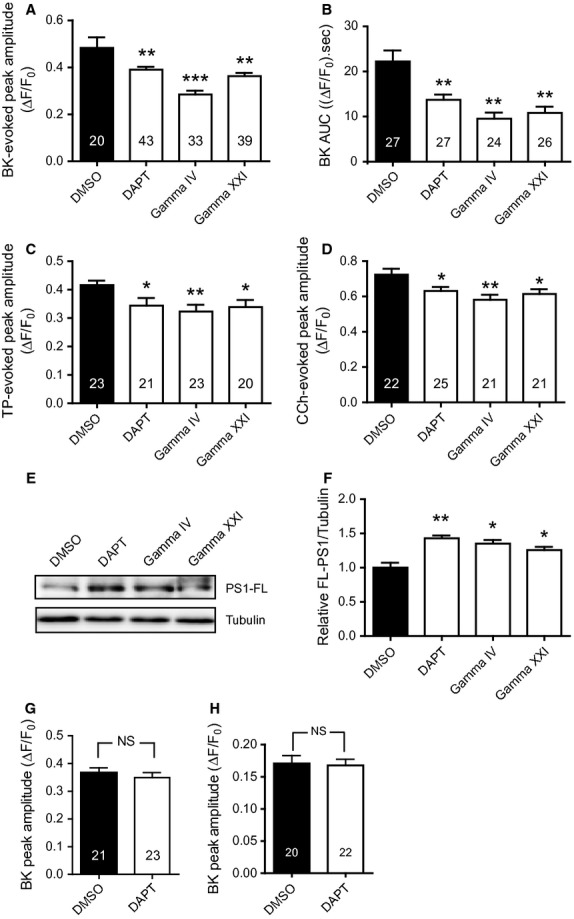
Attenuated calcium release from ER after treatment of HEK293 cells with γ-secretase inhibitors (**A**) Average peak amplitude and (**B**) the area under the curve (AUC) of BK-evoked calcium release and (**C**) the average peak amplitude of TP- and (**D**) CCh-evoked calcium release from ER are significantly reduced in HEK293 cells treated with γ-secretase inhibitors for 24 hrs. DAPT, Gamma IV and Gamma XXI were used respectively at 10 μM, 2.7 μM and 300 nM concentration (*P < 0.05, **P < 0.01 and ***P < 0.001). (**E**) Increase in PS1-FL (holoprotein) levels were detected by western blot in HEK293 cells. (**F**) Densitometric quantification of PS1-FL band intensities normalized to loading control Tubulin in (**E**); (*n* = 3). 10 μg of protein lysate was loaded into each lane on the gel (*P < 0.05 and **P < 0.01). Average peak amplitude of BK-evoked calcium response in (**G**) wild type PS1- and (**H**) PS1-D385N-overexpressing HEK293 cells is unchanged after treatment with DAPT for 24 hrs (NS: Non-significant).

Next we investigated the effect of acute γ-secretase inhibition on the ER calcium release. After treatment of HEK293 cells for 24 hrs with three different γ-secretase inhibitors, we measured BK-evoked calcium responses. Treatment with each of the three γ-secretase blockers, resulted in significantly lowered BK-evoked calcium response compared to vehicle treated controls. The peak amplitude for BK-evoked calcium release for cells treated with DAPT, Gamma IV and Gamma XXI were respectively 80 ± 2.5%, 58 ± 3.3% and 75 ± 2.8% of that for DMSO-treated cells (Fig. 2A). As another parameter proportional to the amount of released ER calcium, the area under the curve (AUC) of BK-evoked calcium responses were calculated. Following a similar trend, the AUC for cells treated with DAPT, Gamma IV and Gamma XXI were respectively 61 ± 5.1%, 42 ± 6.1% and 48 ± 6.1% of DMSO-treated cells (Fig. 2B). Likewise, the amplitude of TP- and CCh-evoked calcium responses were attenuated in γ-secretase inhibitor treated HEK293 cells (Fig. 2C and D). However, treating wtPS1- and PS1-D385N-overexpressing cells with DAPT did not further potentiate the reduction of BK-evoked calcium release (Fig. 2G and H). γ-secretase inhibitors were used at concentrations which were previously described to inhibit the γ-secretase activity. Based on the literature that suggest γ-secretase activity is required for endoproteolysis of PS [33], we postulated that the treatment with γ-secretase blockers might inhibit the endoproteolysis of PS1 itself as well. Despite the expected faint expression of PS1 holoprotein at endogenous levels which could only be detected at longer exposures, we could indeed detect a modest increase in the PS1 holoprotein levels upon 24 hrs treatment of cells with each of the three γ-secretase inhibitors by western blotting (Fig. 2E). Treatment with DAPT, Gamma IV and Gamma XXI respectively led to 48 ± 4.1%, 45 ± 5.4% and 34 ± 4.6% increase in detected PS1 holoprotein levels by western blot (Fig. 2F).

### Effect of PEN-2 knockdown on ER calcium release

**Fig. 3:**
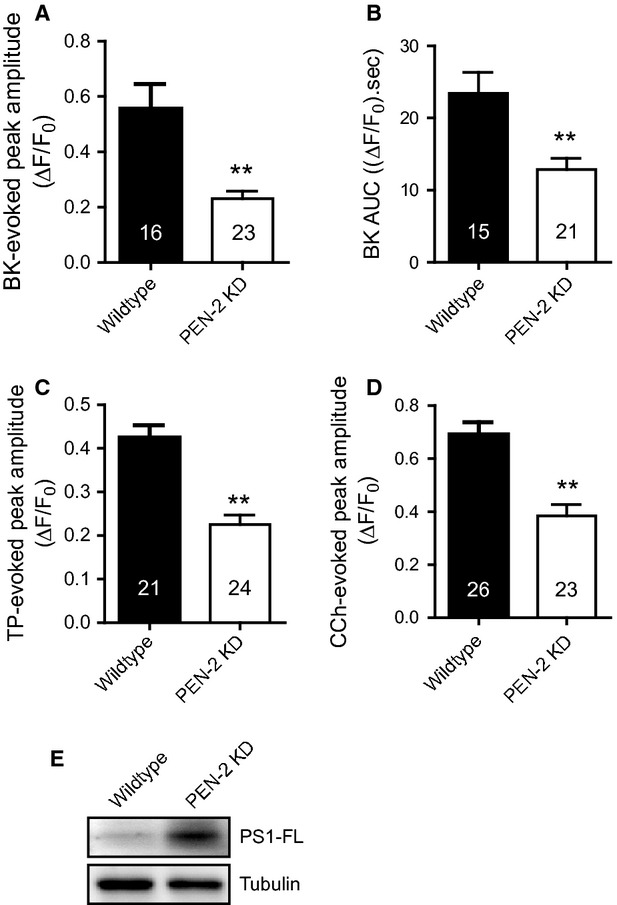
Attenuated calcium release from ER in PEN-2 KD cells (**A**) Average peak amplitude and (**B**) the area under the curve (AUC) of BK-evoked calcium release and (**C**) the average peak amplitude of TP- and (**D**) CCh-evoked calcium release from ER are significantly reduced in PEN-2 KD cells compared to wild type HEK293 cells (**P < 0.01). (**E**) Increased PS1-FL (holoprotein) levels detected by western blot in PEN-2 KD cells. 10 μg of protein lysate was loaded into each lane.

Presenilin enhancer 2 (PEN-2) is a key regulatory component of the γ-secretase complex [34]. PEN-2 is necessary for the proper assembly of active γ-secretase complex and the knockdown of PEN-2 is associated with deficiency in PS endoproteolysis leading to stabilization and accumulation of PS holoprotein [35, 36]. Here we used RNA interference-mediated PEN-2 stable knockdown (PEN-2 KD) in HEK293 cells [28]. The cell line was previously characterized by Prokop and colleagues. In this cell line, increased PS1 holoprotein levels accompanying the downregulation of PEN-2 was demonstrated [28]. The interaction of PS holoprotein with PEN-2 is a key step for PS holoprotein to adopt a conformation which allows its endoproteolytic cleavage [28]. The PS1 holoprotein increase was confirmed in PEN-2 KD cells (Fig. 3E). Here we report that BK-evoked calcium release is attenuated in PEN-2 knockdown cells relative to wild type controls, similar to the observation made in PS1 overexpressing cells. The peak amplitude and the area under the curve in PEN-2 KD cells were respectively 42 ± 4.9% and 55 ± 6.6% of those for wild type cells (Fig. 3A and B). Similarly, the amplitude of TP- and CCh-evoked calcium responses were also decreased in PEN-2 KD cells (Fig. 3C and D).

### PS1 holoprotein in brains of FAD-PS1 patients

**Fig. 4:**
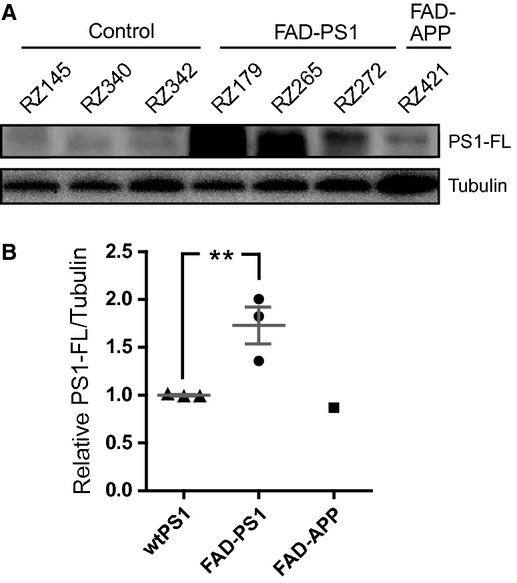
Elevation of PS1 holoprotein levels in frontal cortices of FAD-PS1 patients. (**A**) Significant increase in PS1-FL (holoprotein) levels was detected by western blot in postmortem frontal cortex samples from FAD-PS1 cases (RZ179, RZ265 and RZ272), but not in a FAD-APP case (RZ421), compared to control cases (RZ145, RZ340 and RZ342). 15 μg of brain homogenate was loaded in each lane on the gel (**P < 0.01). (**B**) Densitometric quantification of PS1-FL band intensities normalized to loading control Tubulin in (**A**).

To assess the physiological relevance of the finding that the accumulation of PS holoprotein is associated with the attenuated ER calcium release in the context of AD pathogenesis, we compared the PS1 holoprotein levels in the postmortem frontal cortices of FAD patients relative to non-demented control cases. As expected, the amount of PS1 holoprotein level in control individuals was relatively low. However we observed on average 1.7-fold significant increase in PS1 holoprotein levels in three FAD-PS1 patient cases. In contrast, the level of PS1 holoprotein in a FAD-APP patient was comparable to the controls (Fig. 4A and B). Table 1 summarizes the patient data from which the samples were collected.

**Table 1:** Human frontal cortex postmortem brain materials examined for PS1 holoprotein levels

Sample number	Diagnosis	Age (years)	Sex	Postmortem interval (hours)	Braak & Braak stage
RZ145	Control	85	F	20	I
RZ340	Control	54	M	9.5	0
RZ342	Control	83	F	22	II
RZ179	PS1 Leu286Val	57	M	44	VI
RZ265	PS1 Leu174Arg	57	F	34	VI
RZ272	PS1 Ile143Thr	39	M	7	VI
RZ421	APP Thr714Ile	49	M	48	VI

## Discussion

Here we report that the conditions causing an enhancement in the amount of PS1 holoprotein, result in attenuated calcium release from the ER. Only very little is known about the exact mechanism of PS holoprotein endoproteolysis. Despite the controversial findings, some FAD-PS mutations have been shown to impair the PS endoproteolysis leading to accumulation of PS holoprotein primarily on the ER membrane [26, 27].

Although there exists evidence in favour of FAD-PS mediated ‘ER calcium overload’ theory [37], the results presented here and several other studies show that the FAD-PS and to a lesser extent wild type PS lead to either attenuated or unchanged ER calcium [15, 16, 21, 23, 25, 38–41]. The reason behind such discrepancies is not completely clear. However, in comparing such data one has to critically discriminate between the FAD-PS mediated hyperactivity of InsP_3_R or RyR channels from ‘ER calcium overload’. Those exaggerated calcium responses may simply be a result of enhanced receptor gating and/or density [15, 16, 18, 19], increased basal phospholipase C (PLC) activity and the consequent overproduction of InsP_3_ molecule [42, 43], or a combination of those, while being independent from ER calcium content. While previous studies have demonstrated the biochemical interaction between PS1 and InsP_3_R [16], it is established that the overxpression of FAD-PS does not alter the abundance of InsP_3_ receptors [44]. Notably, we have also observed augmented carbachol (CCh)-evoked calcium responses in FAD-PS1 mutant bearing cells (Fig. 1G and H). While calcium release upon stimulation with both BK and CCh is associated with G-protein coupled receptor (GPCR) activation and InsP_3_ generation, in spite of sharing the same calcium pools, such differential effects between BK- and CCh-evoked calcium responses have been previously described in airway smooth muscle and neuroblastoma cells and proposed to be regulated by differences in the specific PLC involvement and the expression of muscarinic receptors [45, 46]. Furthermore, activation of muscarinic receptors with CCh is associated with remarkably higher InsP_3_ generation as to the activation of bradykinin receptors by BK [47]. Therefore it is possible that in CCh-evoked calcium release experiments (but not BK-evoked), the contribution of FAD-PS1 mediated hyperactivity of InsP_3_ receptors [15, 16], can mask the lowered ER calcium content. Although CCh has been reported to induce calcium release also from RyRs in an InsP_3_R-dependent manner [48], due to extremely low expression levels of RyR in HEK293 cells, the contribution of RyRs in the observed differential effects is rather unlikely [49]. Further work is needed to comprehensively elucidate the differences between BK- and CCh-evoked calcium responses in the context of FAD-PS expression.

It has been proposed that the holoprotein form of PS can function as passive calcium leak channels on the ER membrane allowing the leakage of calcium into the cytoplasm [12, 13, 50]. Majority of FAD-PS mutations lead to loss of this function, some do not affect and others even cause a further gain of this function [51]. Independently from how FAD-PS mutations modulate the leak activity, it is plausible that the increase in PS holoprotein amounts may directly increase the degree of passive calcium leakage from ER to cytoplasm. This can explain our observation that conditions increasing the amount of PS1 holoprotein result in reduced ER calcium. Constant enhanced calcium leakage from ER into cytoplasm as a result of PS holoprotein accumulation will in turn affect the calcium equilibrium between ER and cytosplasm in which the reached steady state of ER calcium level is relatively low. Similar to FAD-PS1 mutants, a loss of function mutant for γ-secretase activity (PS1-D385N) [32] showed reduced calcium release from ER. This observation is in line with another finding showing that γ-secretase cleavage activity is dispensable for the reduction of ER calcium levels, demonstrated using a different γ-secretase inactive mutant (PS2-D366A) [39]. The PS-mediated attenuation of ER calcium release seems to be a specific effect and not caused by ER stress or protein overload, since the overexpression of rAChRα1 which also accumulates at the ER [52], did not alter the amplitude of BK-evoked calcium release.

We demonstrated that treatment with γ-secretase inhibitors lowers the magnitude of calcium release from ER in HEK293 cells. A similar finding has been previously described by others as well [53]. However here we propose an alternative explanation for this phenomenon which is independent from γ-secretase substrate cleavage activity and the suggested functional role for APP intracellular domain (AICD) in calcium signalling [53]. We reveal convincing evidence that treatment of HEK293 cells with γ-secretase blockers results in enhanced PS1 holoprotein levels. This is not very surprising given that γ-secretase blockers may also simultaneously inhibit the PS autocatalytic activity. The elevated PS1 holoprotein levels would in turn lead to enhanced leakage of calcium from ER into cytosol and consequently lowered ER calcium content. Further support for this hypothesis comes from the work of Fukumori and colleagues which nicely demonstrate that the PS holoprotein endoproteolysis is indeed autolytic [33]. Although the detected increase in PS holoprotein was only marginal, in view of extremely low levels of endogenously expressed PS holoprotein as well as the tremendous calcium gradient between ER and cytosol [10], even such minor but sustained enhancements in passive calcium leakage through PS holoprotein may heavily impact the calcium equilibrium between ER and cytosol. In both wtPS1 and PS1-D385N overexpressing cells, treatment with DAPT did not further reduce the amplitude of BK-evoked calcium release. These findings are indeed in line with our hypothesis, since PS1-D385N mutant is deficient for endoproteolysis and a marginal increase in PS1 holoprotein levels caused by DAPT treatment in wtPS1 cells would be negligible in the presence of constitutive PS1 overexpression which accompany abounding PS1 holoprotein levels. In view of therapeutic applications, the finding that γ-secretase inhibitors can elevate the PS holoprotein levels reflects yet another potential undesirable side-effect associated with the use of γ-secretase inhibitors (apart from unspecifically blocking the processing of several substrates other than APP) which should be taken into consideration [54].

Likewise, we demonstrated that the knockdown of PEN-2 leads to attenuated ER calcium release. Prokop and colleagues have shown that the knockdown of PEN-2 is associated with deficiency in PS1 endoproteolysis and accumulation of PS1 holoprotein [28]. Using the same cell line, here we show reduced BK-, TP- and CCh-evoked ER calcium responses as well. These findings reinforce an inverse correlation between the PS holoprotein levels and the amount of calcium release from ER.

In previous studies utilizing AD patient post-mortem brains, the activation of calcium-dependent proteases and the alterations in the activity and abundance of proteins involved in calcium homeostasis were detected [55–57]. In this study, in spite of limited number of human postmortem brains from FAD-PS1 cases, we reveal convincing indication that the amount of PS1 is upregulated in the brains of patients harbouring different FAD-PS1 mutations. Evidence exists that even in brains of sporadic late-onset AD cases, PS1 protein and mRNA levels and γ-secretase activity are upregulated [58]. Li *et al*. have postulated that presenilin upregulation may contribute to sporadic AD as a risk factor in the context of γ-secretase activity [59]. However, more detailed studies are needed to address whether the disturbed calcium homeostasis associated with PS holoprotein accumulation plays a role in the pathogenesis of late onset sporadic AD cases too. PS holoprotein is quite unstable with a relatively short half-life (∼1.5 hrs) and a rapid turnover [60]. Indeed, FAD-PS1 DeltaE9 mutant was shown to possess relatively higher stability and a longer half-life (∼40 hrs) [60]. Weihl and colleagues have suggested that FAD-PS mutations can alter the stability of PS holoprotein in a cell-type and differentiation-state dependent manner [61]. Given the emerging roles for PS outside of γ-secretase complex, increased stability and/or accumulation of PS holoprotein may have direct implications in pathophysiology of AD, particularly through their involvement in disruption of ER calcium homeostasis.

The disturbances in ER calcium homeostasis have been observed in both familial and sporadic AD cases long preceding the disease hallmarks, *i.e*. senile plaque and tangle pathology [62, 63]. While increasing age is the main risk factor for development of sporadic AD, indications suggest that age-dependent alterations in the calcium homeostasis may contribute to the pathogenesis of sporadic AD as well [64]. Moreover calcium dysregulation plays a key role in synaptic failure and neuronal loss [65]. Notably, the latter pathological events correlate best with the stages of dementia [66]. Therefore better understanding the underlying mechanisms responsible for the disruption of ER calcium homeostasis would be valuable in development of therapies targeting calcium dyshomeostasis as an early event in AD pathogenesis.

There are controversies in the literature as to how FAD-PS mutations alter ER calcium handling. These variations were mainly attributed to differences in methodologies used, different cells types and mutations. However based on the results here, we suggest that in the experimental setups where either wild type or mutant PS forms are overexpressed, possible differences in the PS expression levels may potentially give rise to the inconsistencies between independent studies. This point becomes even more critical taking into account that the expression level of PS holoprotein is quite low under physiological conditions. Given the fact that only a fraction of PS can incorporate into γ-secretase complex [67], constitutive overexpression might be suitable for studying the effect of PS mutations on γ-secretase activity. However when FAD-PS-dependent alterations in the ER calcium homeostasis are investigated, overexpression of PS might not fully resemble their role in pathophysiological circumstances. Therefore the use of patient derived FAD-PS cells (*e.g*. fibroblasts, lymphocytes, *etc*.) which express PS holoprotein at endogenous levels may be more appropriate. On the other hand, since FAD-PS mutations alter the stability of PS holoprotein in a cell-type and differentiation-state dependent manner [61], calcium homeostasis in periphery might not exactly correspond to the FAD-PS-mediated disruption of calcium homeostasis in the brain. Despite the mentioned drawbacks associated with the use of overexpression and secondary cell models, HEK293 cells were suitable for the purpose of this study. Regulation of calcium homeostasis in neurons is a very complex mechanism which is tightly controlled by the functions of multiple molecular elements. The existence of compensatory mechanisms which by masking studied effect can efficiently restore balanced calcium homeostasis makes the manipulation of calcium signalling in neurons for studying cause-effect relationships rather challenging. By contrast, in simpler cell models (*e.g*. HEK293 cells), in shortage of such efficient compensatory mechanisms, directly assessing the effect of PS1 holoprotein accumulation on ER calcium homeostasis was more readily possible.

Taken together our results reinforce the notion that the accumulation of full length forms of PS (as a result of *e.g*. impaired PS autoendoproteolysis) result in reduced ER calcium content. Therefore future studies are necessary to examine whether the adverse FAD-PS-mediated effects on the functions of several ER calcium handling elements, including described loss of PS calcium leak channel activity [13], enhanced InsP_3_ [15, 16] and RyR channel activity [17, 18] and abundance [19, 68] could be secondary mechanisms to compensate for the PS holoprotein-associated attenuation of ER calcium load.
